# Age Differences in Age Perceptions and Developmental Transitions

**DOI:** 10.3389/fpsyg.2018.00067

**Published:** 2018-02-01

**Authors:** William J. Chopik, Ryan H. Bremner, David J. Johnson, Hannah L. Giasson

**Affiliations:** ^1^Department of Psychology, Michigan State University, East Lansing, MI, United States; ^2^Department of Psychology, University of St. Thomas, Saint Paul, MN, United States; ^3^Department of Psychology, Stanford University, Stanford, CA, United States

**Keywords:** age perceptions, developmental transitions, project implicit, age differences, middle age, older adulthood

## Abstract

Is 50 considered “old”? When do we stop being considered “young”? If individuals could choose to be any age, what would it be? In a sample of 502,548 internet respondents ranging in age from 10 to 89, we examined age differences in aging perceptions (e.g., how old do you feel?) and estimates of the timing of developmental transitions (e.g., when does someone become an older adult?). We found that older adults reported older perceptions of aging (e.g., choosing to be older, feeling older, being perceived as older), but that these perceptions were increasingly younger than their current age. The age to which individuals hope to live dramatically increased after age 40. We also found that older adults placed the age at which developmental transitions occurred later in the life course. This latter effect was stronger for transitions involving middle-age and older adulthood compared to transitions involving young adulthood. The current study constitutes the largest study to date of age differences in age perceptions and developmental timing estimates and yielded novel insights into how the aging process may affect judgments about the self and others.

“I will never be an old man. To me, old age is always 15 years older than I am.”

– Francis Bacon

## Introduction

Walking through a birthday card aisle offers plenty of reminders about how aging is something to avoid. Life begins at 40. Fifty is the new 30. Although these cards often represent tongue-in-cheek ways of helping the recipient feel better about aging, very little is known about how both perceptions of age and estimates of the timing of developmental transitions differ by age. Is 50 “old”? When do we stop being “young”? If individuals could choose to be any age, what age would they be? The current study examines age differences in aging perceptions (e.g., how old do you feel?) and estimates of the timing of developmental transitions (e.g., when does someone become an older adult?).

## Perceptions of aging

In the current study, we operationalize aging perceptions as evaluations individuals tie to different ages by reporting (a) the age they would like to ideally be, (b) the age they feel like, (c) the age they hope to live until, and (d) how old other people think they are. To date, most research has focused on lifespan differences in and consequences of (b), which researchers refer to as *subjective age* (Kleinspehn-Ammerlahn et al., 2008). There is a large literature documenting the antecedents and consequences of subjective age that highlights the roles of subjective health, age-group reference effects, gendered experiences, and aging attitudes (see Montepare, 2009). The preponderance of research suggests that adults tend to report feeling younger than their chronological age (e.g., up to 20% younger; Rubin and Berntsen, 2006) and this effect increases with age. Reporting a younger subjective age is associated with a wide variety of benefits for health and well-being (Montepare and Lachman, 1989; Kotter-Grühn et al., 2009; Mock and Eibach, 2011). However, feeling younger is not the only aging perception that changes across the lifespan. There are also corresponding shifts toward youth for how old people think they look, what their interests are, and the activities they like to engage in (Kastenbaum et al., 1972).

Why does a shift toward affiliating with youth happen more as people age? Insights from the age-group dissociation effect provide a potential explanation (Weiss and Lang, 2012). In short, people try to psychologically dissociate themselves from stigmatized groups (i.e., older adults). When stigmatized outgroups are salient, people engage in avoidance-oriented behavior. Motivations underlying the age-group dissociation effect can be identified in the evolutionary psychology literature (North and Fiske, 2012). For example, inclusive fitness cues motivate individuals to prefer helping younger relative to older adults in times of need (Burnstein et al., 1994). Likewise, individuals often associate older adults with weakness, resource waste, and possible exposure to infectious disease—all of which lead to higher levels of stigma (Jensen and Oakley, 1980; Kurzban and Leary, 2001; Duncan and Schaller, 2009).

Previous research has identified many antecedents and consequences of the age-group dissociation effect. For example, openness to experience and less traditional gender ideologies might be protective factors for well-being among individuals undergoing difficult and uncertain age transitions (Weiss et al., 2012). Further, age group dissociation can protect individuals from the deleterious effect that negative age stereotypes have for older adults' self-esteem (Weiss et al., 2013). Some of the distancing techniques that older adults employ include identifying with middle aged adults and even directing their attention away from other older adults (Weiss and Freund, 2012).

In sum, older adulthood is an identity that carries significant stigma (Levy and Banaji, 2002), and individuals become increasingly closer to assuming this stigmatized identity as they age. When people become older adults, they could view themselves as becoming part of a group to which they have held negative attitudes toward their whole life. In general, individuals are motivated to create psychological and physical distance between themselves and stigmatized outgroups (e.g., Cesario et al., 2010). In this case, one way in which people can enhance this distance is to identify with younger age groups, whether that be through selectively reporting feeling younger than they are, reporting that others perceive them as being younger, or choosing a younger ideal age to be (Blau, 1956; Heckhausen and Krueger, 1993; Weiss and Lang, 2012). By extension, adolescents might report a relatively *older* subjective age given their desire to affiliate with a more desirable group (e.g., young adults; Galambos et al., 2005, 2009).

There is also a sense that an individual's reference group changes as they age. For example, younger adults who compare themselves to other younger adults are unlikely to distort their subjective age because young adults are not a stigmatized group (Galambos et al., 2009). However, adolescents and older adults share a motivation to identify with more highly regarded age groups and thus distort or shift their perceptions of aging. Nevertheless, even in the context of age-group dissociation, older adults, being closer to the end of their lives, may push their ideal life expectancy to an older age from a motivation toward self-preservation (Karni and Schmeidler, 1986; Brown et al., 1999). Indeed, multiple studies have shown that older adults increase their ideal-age-to-live-until as a way of elongating horizons in the face of mortality (Mirowsky, 1999; Lang et al., 2007). Some researchers have also hypothesized that older adulthood might serve as a reminder of mortality—triggering protective, life-elongating defense mechanisms to mitigate the anxiety that arises from these reminders (Maxfield et al., 2010; Chopik, 2017).

Given research on the age-group dissociation effect in which people try to psychologically distance themselves from older adults, we hypothesized that, compared to younger adults, older adults would report (a) ages that they ideally would like to be that are older, albeit ages that are increasingly younger than their chronological age, (b) older subjective ages, albeit ages that are increasingly younger than their chronological age and (c) being perceived by others as older, albeit increasingly younger than their chronological age. Younger adults will report age perceptions closer to their chronological age because younger adults are not stigmatized in the same way that older adults are. Given research on self-preservation and mortality reminders, we also hypothesized that older adults would report an older ideal age to live until.

## Developmental transitions

The exact age at which older adulthood starts is hotly debated in the social and developmental psychology literature. Different fields and researchers use different indices—biological indices (López-Otín et al., 2013), cognitive indices (Hasher and Zacks, 1988), expected years left to live (Sanderson et al., 2017), or historical standards (Roebuck, 1979)—for determining what makes someone old. Like aging perceptions, the perceived timing of developmental transitions depends on where individuals are in the life course. For example, older adults tend to report that older adulthood happens at a later age relative to younger adults (Barrett and Von Rohr, 2008; Barrett and Toothman, 2016). Indeed, a recent Pew survey further replicated this effect, showing adults 18–29 believe that a person becomes old at age 60, whereas middle-aged respondents believe that a person becomes old at age 72; respondents aged 65 and older believed that a person becomes old at age 74 (Taylor et al., 2009). Longitudinal studies of middle-aged adults suggest a similar effect—that individuals “elongate” the age range that one is considered a middle-aged adult as they live through this period themselves (Kuper and Marmot, 2003). Like research on the protective functions of subjective age, perceiving the middle age period as longer is associated with a host of positive health outcomes, including a lower risk of hypertension, diabetes, functional limitations, heart disease, and better recovery from illnesses (see Barrett and Toothman, 2014, 2016).

Unfortunately, work on normative perceptions of age transitions has several limitations. For example, most studies examine only one age group's perceptions of developmental transitions (Barrett and Von Rohr, 2008) or ignore certain groups (e.g., middle-aged adults) entirely by comparing only extreme groups of younger and older adults (Cohen, 1983; Freund and Isaacowitz, 2013). Further, research on estimates of developmental transitions have focused exclusively on instructing participants to report the perceived age of either the average middle-aged (Kuper and Marmot, 2003) or older adult (Barrett and Toothman, 2016). Less is known about younger developmental transitions and how perceptions of these transitions differ by age. Do transitions from childhood to young adulthood show similar age differences, such that older adults give older estimates even for transitions that are less socially stigmatized? In the current study, we address these limitations by employing a large sample of adults (*N* = 250,000 +) ranging in age from 10 to 89 to examine age differences in estimates of developmental transitions (i.e., childhood to young adulthood, young adulthood to adulthood, adulthood to middle age, and middle age to older adulthood).

To date, we know little about age differences for these perceptions of younger developmental transitions. Drawing on some of the same principles from age-group dissociation effects, we hypothesized that age differences for these younger transitions would be smaller in magnitude compared to older transitions. Given that older transitions (e.g., becoming a middle-aged or older adult) are more stigmatized, the age-group dissociation effect should be large for these transitions, given that individuals try to psychologically distance themselves more from these groups. As such, middle-aged and older adults should “push” these transitions further into the future, giving increasingly older age estimates to make themselves appear to be relatively younger. At the same time, the age-group dissociation phenomenon also suggests that middle-aged and older adults should “pull” younger developmental transitions toward their current age. In distancing themselves from stigmatized groups, people are motivated to identify with groups that affirm positive aspects themselves (Abrams and Hogg, 1988). Thus, we hypothesized that older adults would report higher estimates for early life transitions as well. Younger adults are will simply use their cultural knowledge to estimate these transitions. In other words, they will be unlikely to be motivated to shift their developmental timing estimates because of the distance from stigmatized age transitions that their chronological age affords them. Thus, younger developmental transitions should be observed among younger adults when compared to older adults' estimates.

## The current study

In the current study, we examined age differences in perceptions about aging and estimates for developmental transitions. Perceptions about aging were operationalized as (a) what age participants would choose to be, (b) how old participants feel, (c) what age participants would like to live until, and (d) how old other people think participants are. We expected that older adults would report older perceptions compared to younger adults, albeit younger perceptions relative to their current age (e.g., younger subjective and ideal ages; Kleinspehn-Ammerlahn et al., 2008). We expected younger adults to provide estimates close to their chronological age considering that they are part of a valued age group. Older adults were expected to report an older age they would like to live until. We also expected older adults to report older age estimates for developmental transitions in an effort to identify more with younger adults, which they consider a more valued age group (Weiss and Lang, 2012). Specifically, older adults will perceive both younger transitions and older transitions as happening at later ages to make themselves feel relatively younger. Finally, because older adults are a stigmatized group, we expected this shift to be particularly true of transitions into older adulthood relative to transitions into younger age groups. Younger adults were not expected to shift the timing of developmental transitions given their distance from stigmatized age groups. To test these hypotheses, we ran regression analyses predicting the raw ages that participants provided for age perceptions and developmental transitions.

## Methods

### Participants and procedure

Participants were 502,548 individuals (69.1% Female) from the Project Implicit Demo Site, a website that hosts studies on the Implicit Association Test (data and materials are available at osf.io/cv7iq/). No targeted recruitment was undertaken. People interested in implicit bias navigate to the website and complete a measure of implicit bias in exchange for personalized feedback. Thus, the entire sample is one of convenience—people freely completing measures about aging with no expectation of compensation.

Data were collected from September 2006 to December 2015, the entire time window for which the questions were posted and data are available. The overall sample ranged in age from 10 to 89 (*M* = 26.88 years, *SD* = 12.14 years); the median level of education was some college. Self-reported race/ethnicity was 69.3% Caucasian, 9.8% Hispanic, 7.7% African American, 7.2% Asian, and 6.1% Mixed/Other ethnicities. Despite the sample being relatively young, each decade of life was well represented (e.g., 10–19 years: 162,601; 20–29 years: 199,565; 30–39 years: 58,866; 40–49 years: 40,837; 50–59 years: 29,517; 60–69 years: 9,490; 70+ years: 1,672). An additional 315,394 participants were excluded from the present analyses because these participants were younger than 10, had missing data on age, or had missing data on all the primary study variables. The majority of respondents were from the United States (85.3%) and the survey was presented entirely in English. Supplementary analyses examining differences between the United States and other countries generally found similar effects. For several methodological and conceptual reasons, cultural comparisons were not part of the current report^1^. We further elaborate on this decision in the Discussion section.

Because the Project Implicit site's primary purpose is to host variants of the Implicit Association Test, we also had data on implicit and explicit age bias. The order of the IAT and one of the two blocks of self-report questions (perceptions about aging or age estimates for developmental transitions) were counterbalanced across participants. Associations between implicit/explicit bias and the variables below are consistent with predictions made from age-group dissociation effect (e.g., greater bias against older adults was associated with younger age perceptions), albeit these associations were small (|0.01| < *r* < |0.22|). As such, we did not include these data in the current report but felt the need to disclose that these data are available. For a review of age differences in implicit and explicit bias using this data set, please see Chopik and Giasson (2017).

Because we analyzed an existing data source, the Michigan State Institutional Review Board considered this research exempt from ethical oversight as it did not constitute human subjects research (IRB# 17-1113).

### Perceptions about aging

A random sample of participants (*N* = 251,496) received four open-ended questions asking which age they would choose to be (“If you could choose, what age would you be?”; hereafter *age choice* in all tables), what age they felt like (“How old do you feel?”; *subjective age*), what age they hope to live until (“To what age do you hope to live?”; *hope to live*), and how old other people think they are (“On average, how old do other people think you are?”; *perceived age*). Participants typed in a numeric age in response to each question. Descriptive statistics for these questions can be found in Table 1.

**Table 1:** Correlations and descriptive statistics among primary study variables.

	**1**	**2**	**3**	**4**	**5**	**6**	**7**	**8**	**9**	**10**
1. Gender										
2. Age	−0.03									
3. Age choice	0.01	0.68								
4. Subjective age	−0.04	0.74	0.57							
5. Hope to live	0.02	−0.02	−0.01	−0.04						
6. Perceived age	−0.08	0.89	0.64	0.73	−0.02					
7. Childhood-young adult transition	0.03	0.11								
8. Young adult-adult transition	0.03	0.13					0.60			
9. Adult-middle age transition	0.08	0.27					0.20	0.36		
10. Middle age-older adulthood transition	0.11	0.30					0.09	0.20	0.63	
*M*	–	26.88	24.26	26.03	89.42	25.09	15.74	22.04	40.13	62.78
*SD*	–	12.14	9.28	10.80	15.73	10.32	3.10	4.17	7.29	9.64

*Ns range from 249014 to 499744. All correlations are significant at p < 0.001. Gender: −1, Male; 1, Female*.

### Age estimates for developmental transitions

A different random sample of participants (*N* = 251,052) received four open-ended questions asking the age at which four developmental transitions occurred. The four transitions were from childhood to young adulthood (“A person moves from being a child to being a young adult at what age?”), from young adulthood to adulthood (“A person moves from being a young adult to being an adult at what age?”), from adulthood to middle-age (“A person moves from being an adult to middle-aged at what age”), and from middle-age to older adulthood (“A person moves from being middle-aged to being old at what age?”). Participants typed in a numeric age in response to each question. Data on the preceding age perception questions were not collected for these individuals. Descriptive statistics for these questions can be found in Table 1.

### Analytic approach

Because of our large sample, there was a concern that many effects would likely be statistically but not practically significant. To address this, we employed an effect size-based approach to interpret our results (Srivastava et al., 2003; Chopik and Edelstein, 2014). As in previous research, we limited our discussion to individual effects that exceed a certain threshold deemed meaningful when using large samples (Δ*R*^2^ ≥ 0.001 and *F*_*change*_ ≥ 25) (Chopik et al., 2013). Further, prior research suggested that the most complex age trends that can be meaningfully interpreted involve cubic patterns (Terracciano et al., 2005). Thus, we tested the linear (age), quadratic (age^2^), and cubic (age^3^) effects of age; we did not test more complex models. Age was centered prior to computing these higher order terms in order to reduce multi-collinearity. Gender was included as a control variable in each model given research on gendered perceptions of what is considered an older adult (Zepelin et al., 1987; Seccombe and Ishii-Kuntz, 1991; McConatha et al., 2003). We initially tested incremental models (i.e., predicting perceptions and age estimates from an individual age term, before adding a more complex pattern) before realizing that in nearly every case (except for two), the inclusion of age^2^ and age^3^ surpassed our effect size threshold. We report the full models for simplicity with individual *F*_*changes*_ for each estimate, but the information for the sequential model testing analysis can be requested from the first author.

## Results

### Preliminary results

Descriptive statistics and intercorrelations among the study variables are given in Table 1,^2^. We limit our discussion to correlations greater than 0.05. As a reminder, we hypothesized that (a) older adults would report older perceptions of aging (e.g., feeling older) and (b) older adults would report older age estimates across developmental transitions.

As an initial confirmation of our hypotheses, older adults reported choosing an older age to ideally live until, feeling older, and being perceived as older compared to younger adults. Older adults reported older age estimates for each of the transitions, also supporting our hypotheses. We more formally modeled non-linear associations in the main analyses. Responses were generally correlated within perceptions and within age estimates. Our hypotheses were largely supported, albeit the effect sizes of each were small.

Finally, women also reported being perceived as younger and perceived the adulthood-middle-age and middle age-older adulthood transitions as occurring later in life.

### Main analyses

Age differences in perceptions of aging and age estimates of developmental transitions can be seen in Table 2. For nearly all variables (except *subjective age*, the *childhood to young adult transition*, and the *young adult to adult transition*), the quadratic effect of age was the best fitting model, based on the *F*_*change*_ cut-off criterion. For the remaining three outcomes, the cubic model was the best fit.

**Table 2:** Regression results for age perceptions and developmental estimates.

	***b***	***SE***	**β**	***t***	***p***	**95% CI: LB**	**95% CI: UB**	***F_*change*_***	**Δ*R*^2^**
**AGE CHOICE**									
Age	0.49	0.002	0.64	266.25	< 0.001	0.49	0.49	70888.63	0.15
Gender	0.29	0.02	0.03	19.52		0.26	0.32	380.94	0.001
Age^2^	0.001	< 0.001	−0.01	18.14		0.001	0.002	329.18	0.001
*F*_(3, 249010)_ = 70178.52, *p* < 0.001. *R*^2^ = 0.46. *Inclusion of the cubic term did not surpass our F*_*change*_ *cut-off* (*F*_*change*_ = 2.97).									
**SUBJECTIVE AGE**									
Age	0.70	0.002	0.79	356.41	< 0.001	0.70	0.70	127026.87	0.23
Gender	−0.14	0.02	−0.01	−8.95	< 0.001	−0.17	−0.11	80.12	0.0001
Age^2^	−0.01	< 0.001	−0.23	−45.80	< 0.001	−0.01	−0.01	2097.18	0.004
Age^3^	< 0.001	< 0.001	0.17	36.92	< 0.001	0.0002	0.0002	1362.73	0.002
*F*_(4, 249734)_ = 74867.02, *p* < 0.001. *R*^2^ = 0.55.									
**HOPE TO LIVE**									
Age	−0.08	0.004	−0.06	−19.41	< 0.001	−0.09	−0.07	376.62	0.002
Gender	0.26	0.03	0.02	7.72	< 0.001	0.20	0.33	59.60	0.0002
Age^2^	0.003	< 0.001	0.05	16.36	< 0.001	0.003	0.003	267.58	0.001
*F*_(3, 248816)_ = 149.94, *p* < 0.001. *R*^2^ = 0.002. *Inclusion of the cubic term did not surpass our* Δ*R*^2^ *cut-off* (Δ*R*^2^ = 0.0003).									
**PERCEIVED AGE**									
Age	0.69	0.001	0.81	564.91	< 0.001	0.69	0.69	319127.22	0.25
Gender	−0.64	0.01	−0.06	−64.66	< 0.001	−0.66	−0.62	4180.60	0.003
Age^2^	0.004	< 0.001	0.10	70.81	< 0.001	0.004	0.004	5014.67	0.004
*F*_(4, 249182)_ = 260655.18, *p* < 0.001. *R*^2^ = 0.81. *Inclusion of the cubic term did not surpass our* Δ*R*^2^ *cut-off* (Δ*R*^2^ = 0.00004).									
**CHILDHOOD-YOUNG ADULT TRANSITION**									
Age	0.04	0.001	0.16	49.38	< 0.001	0.04	0.04	2438.75	0.01
Gender	0.10	0.01	0.03	15.21	< 0.001	0.09	0.12	231.45	0.001
Age^2^	−0.002	< 0.001	−0.16	−21.61	< 0.001	−0.002	−0.002	466.86	0.002
Age^3^	< 0.001	< 0.001	0.11	15.45	< 0.001	0.00003	0.00004	238.62	0.001
*F*_(4, 249572)_ = 1012.43, *p* < 0.001. *R*^2^ = 0.02.									
**YOUNG ADULT-ADULT TRANSITION**									
Age	0.07	0.001	0.19	58.37	< 0.001	0.06	0.07	3406.74	0.01
Gender	0.16	0.01	0.04	18.20	< 0.001	0.15	0.18	331.27	0.001
Age^2^	−0.003	< 0.001	−0.23	−30.57	< 0.001	−0.004	−0.003	934.26	0.004
Age^3^	< 0.001	< 0.001	0.16	23.34	< 0.001	0.0001	0.0001	544.60	0.002
*F_(4, 249223)_ = 1431.74, p < 0.001. R^2^ = 0.02*.									
**ADULT-MIDDLE AGE TRANSITION**									
Age	0.20	0.002	0.33	105.51	< 0.001	0.20	0.20	11132.10	0.04
Gender	0.71	0.02	0.09	46.43	< 0.001	0.67	0.73	2155.50	0.01
Age^2^	−0.002	< 0.001	−0.10	−22.98	< 0.001	−0.002	−0.002	528.28	0.002
*F*_(3, 249028)_ = 7540.34, *p* < 0.001. *R*^2^ = 0.08. *Inclusion of the cubic term did not surpass our F*_*change*_ *cut-off* (*F*_*change*_ = 12.49).									
**MIDDLE AGE-OLDER ADULTHOOD TRANSITION**									
Age	0.29	0.002	0.36	115.65	< 0.001	0.28	0.29	13375.44	0.05
Gender	1.29	0.02	0.12	64.93	< 0.001	1.25	1.32	4215.28	0.02
Age^2^	−0.003	< 0.001	−0.08	−24.38	< 0.001	−0.003	−0.002	594.53	0.002
*F*_(3, 249382)_ = 9653.82, *p* < 0.001. *R*^2^ = 0.10. *Inclusion of the cubic term did not surpass our F*_*change*_ *cut-off* (*F*_*change*_ = 9.21).									

**Figure 1:**
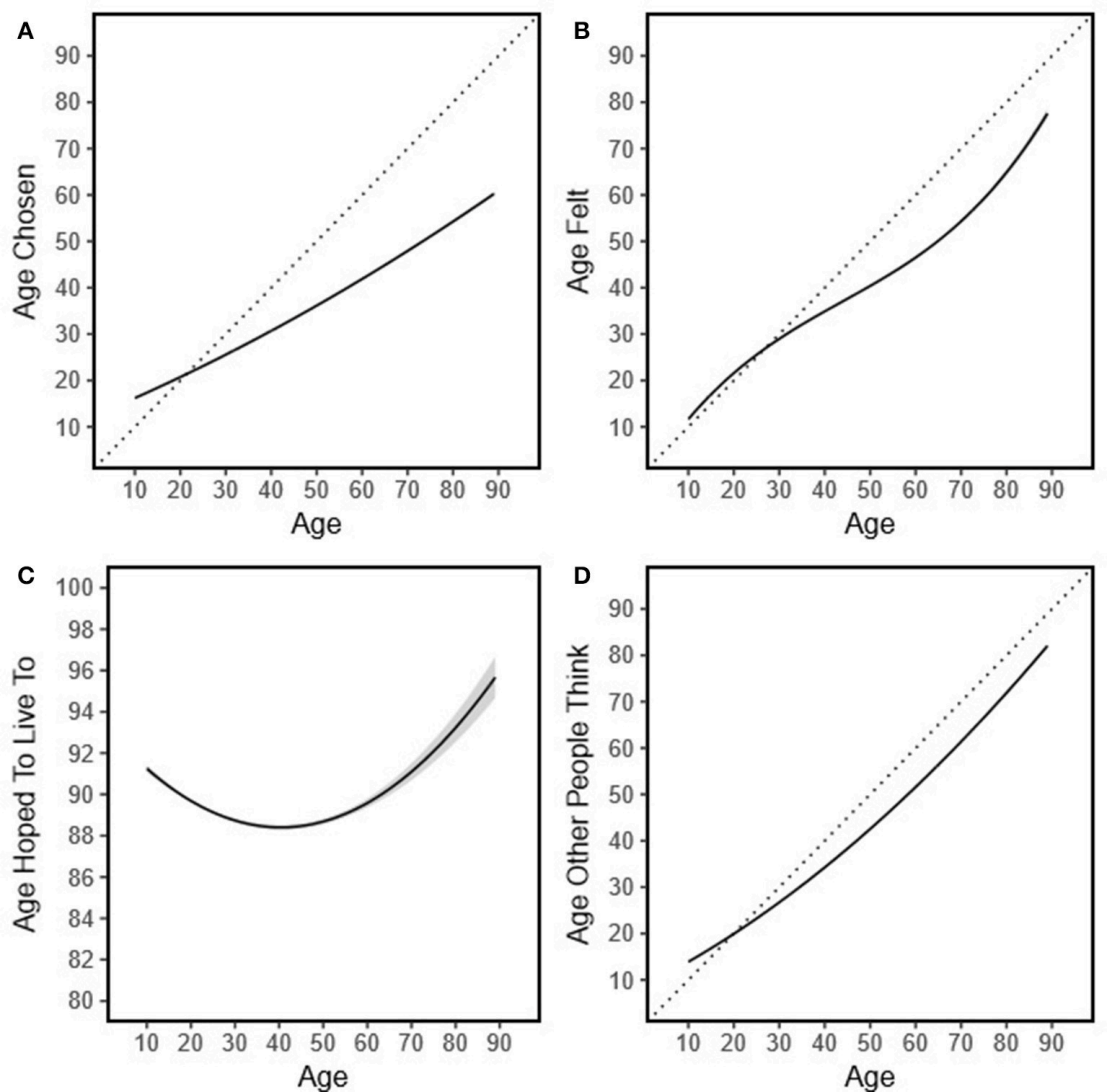
Age differences in age perceptions. The transposed line in **(A,B,D)** represent the identify line for individual age and the age for each dependent variable. **(C)** did not follow the response pattern of **(A,B,D)** with participants reporting that they would like to live to at least 88 yrs old on average; thus, the transposed line is not provided.

As seen in Figures 1A–D, older adults were higher in each age perception compared to younger adults, consistent with the correlational analyses reported above. Specifically, older adults reported older ideal ages, feeling older, and being perceived as older. Worth noting, in each of these cases, each of these three age evaluations were lower than participants' actual ages, particularly among older adults^3^. An interesting quadratic pattern emerged with respect to the age to which participants hoped to live, which was masked in the above correlational analyses (see Figure 1C). After age 40, the age people hope to live to dramatically increased.

**Figure 2:**
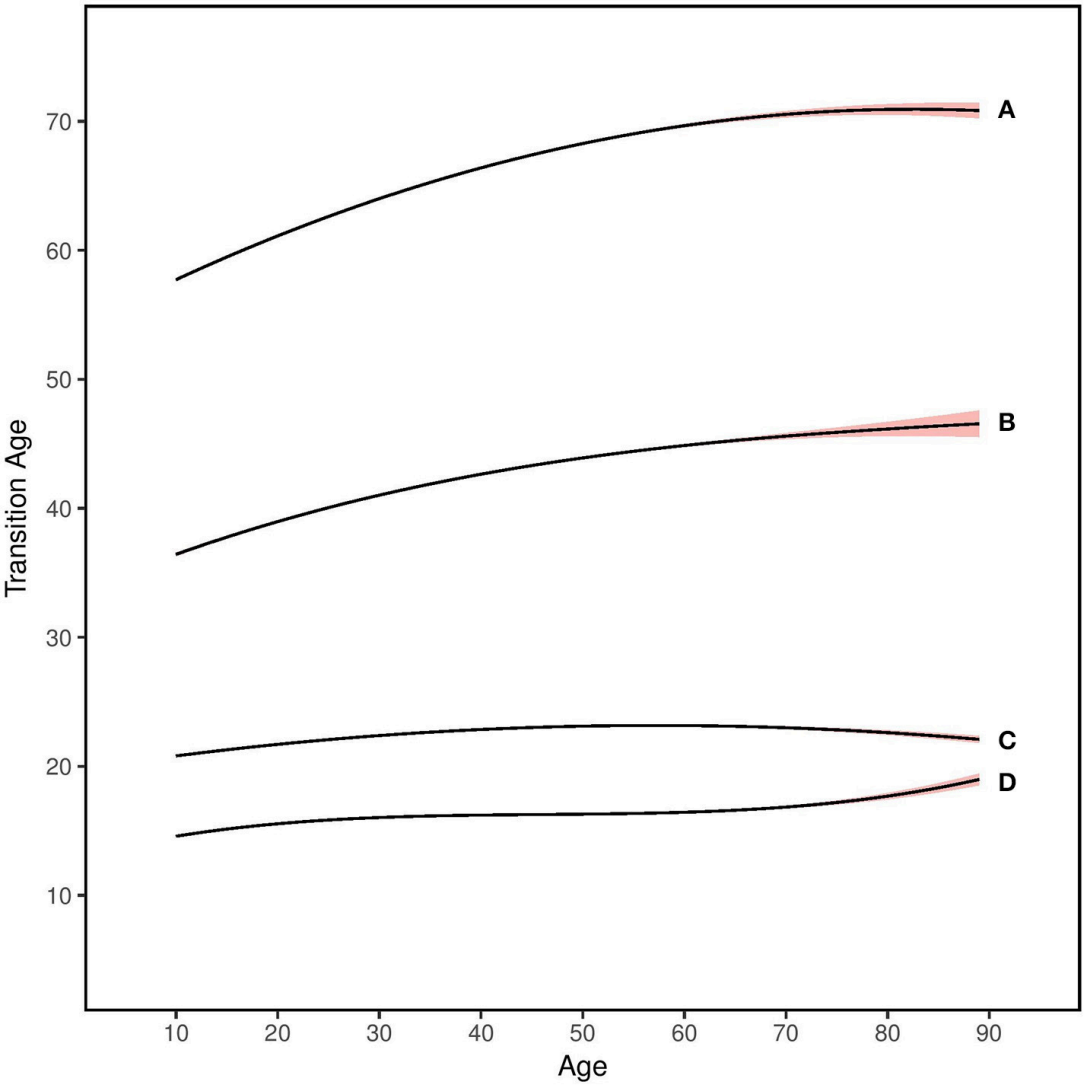
Age differences in developmental transitions for **(A)** middle-age to older adulthood, **(B)** adulthood to middle-age, **(C)** young adulthood to adulthood, and **(D)** childhood to young adulthood. Shaded colors represent 95% confidence intervals.

As seen in Figure 2, older adults gave older age estimates for both younger and older developmental transitions. This shift was especially apparent for older transitions, as seen in the linear effect of age being smaller for the childhood-young adult transition (β = 0.16) and larger for the middle age-older adulthood transition (β = 0.36).

## Discussion

In the current study, we examined age differences in perceptions of aging and estimates for developmental transitions. Older adults reported perceiving themselves as older, but these perceptions were increasingly younger than their current age. We also found that older adults placed the age at which developmental transitions occurred later in the life course. This latter effect was stronger for transitions involving middle-age and older adulthood compared to transitions involving young adulthood. The current study constitutes the largest study to date of age differences in age perceptions and developmental timing estimates and yielded novel insights into how the aging process may affect judgments about the self and others.

The observed age differences in age perceptions and developmental estimates align well with existing research on how our attitudes toward older adults affect judgments about ourselves and others. As people age, they become increasingly closer to identifying with a stigmatized group (i.e., older adults). As a result, people engage in efforts to psychologically distance themselves from older adults. One way in which people do this is to give younger perceptions of aging—reporting that they would choose to be younger, that they feel younger, and that people think they are younger than their current age. Older participants gave older desired lifetimes compared to younger adults, which is consistent with individual's motivations toward self-preservation and defensive reactions toward mortality reminders (Lang et al., 2007; Maxfield et al., 2010). These desired lifetime effects among older adults were reflected in other patterns as well. For example, the association between subjective age and ideal age to live until was higher for older and middle-aged adults (*r*s = −0.08; see Supplementary Materials) compared to younger adults (*r* = −0.04). In other words, older adults who tend to report relatively younger subjective ages also tend to report an older age they would like to live until, suggesting that the defensive processes of the age-group dissociation effect and self-preservation might work hand-in-hand in middle-age and older adulthood. However, future longitudinal research that directly measures these mechanisms can more appropriately document the time scale and process through which these perceptions of aging change across the life span. Older people also shift the timing of developmental transitions to later ages, pulling younger transitions toward their current age and pushing older transitions away from their current age, thus making themselves feel relatively younger. Altogether, our results converge with insights provided by the age-group dissociation effect that individuals avoid identification affiliation with stigmatized groups (Weiss and Lang, 2012).

In the current study, we examined normative age differences in age perceptions and developmental timing. However, a great deal of research is dedicated to experimentally inducing the mechanisms that lead to many of these age differences. Is there evidence for the malleability of age perceptions? Are there ways of counteracting negative perceptions about aging? The vast majority of studies on aging perceptions feature manipulations that increase the salience of negative aging stereotypes (Levy and Banaji, 2002; Levy and Myers, 2004; Levy and Schlesinger, 2005; Levy, 2009). The salience of negative information about aging is often used to induce the age-group dissociation effect (Weiss and Freund, 2012; Weiss and Lang, 2012; Weiss et al., 2013). Few studies have examined how instructing individuals to acknowledge the positive aspects of aging might reduce stereotypes and the age-group dissociation effect. In one exception, Levy et al. (2014) developed an intervention that trained individuals to pair positive words with older adults in an effort to change their implicit associations. In a sample of 100 older adults, they found that enhancing positive associations with aging was associated with more positive age stereotypes, more positive perceptions about aging, and improved physical functioning. However, an explicit intervention in which participants were instructed to “imagine a senior citizen who is mentally and physically healthy” was ineffective for changing participants' attitudes. Unfortunately, few comprehensive and well-powered tests of the extent to which different interventions to reduce age bias and negative age perceptions currently exist (Braithwaite, 2002; Christian et al., 2014). Parallel efforts to reduce other types of bias (e.g., race bias) using existing bias-reduction interventions suggest that the literature's current interventions have very small effects on bias, rarely change explicit behavior, and almost never persist over time (Lai et al., 2013, 2014, 2016). Future research can more adequately test different interventions for changing age perceptions and tailors these interventions to maximize effectiveness in different age groups.

## Limitations

The current study used a large developmentally diverse sample and included measures of both age perceptions as well as estimates of developmental transitions. Nevertheless, there are some limitations worth acknowledging.

Most notably, the cross-sectional nature of the data limits our ability to make causal and developmental claims about how these age perceptions change across the life span. Fortunately, there is both experimental (Weiss and Lang, 2012) and longitudinal work (Kuper and Marmot, 2003) suggesting that the aging process and age stereotypes are the main predictors behind the differences in age perceptions and developmental estimates that we observed in the current study. Nevertheless, future work can examine these questions both experimentally and longitudinally to further strengthen a developmental interpretation. Further complicating our interpretation is that there are likely cohort differences in the link between age perceptions and many important outcomes (e.g., Barrett and Toothman, 2014). Attitudes and expectations about older adults and developmental timing likely change over time and across generations, as they do toward other sociodemographic groups (Zepelin et al., 1987; Westgate et al., 2015). Future studies should adequately disentangle age, period, and cohort effects to examine how perceptions about aging change at different levels and time scales within a broader population.

Another limitation is that our sample consisted of individuals primarily from the United States. This is an important limitation as there is substantial variability in attitudes toward older adults and aging found across cultures (Löckenhoff et al., 2009; North and Fiske, 2015). Unfortunately, several methodological restrictions prevented our ability to systematically study cultural variation in many of our effects. For example, many of the sample sizes of individual countries were very small (i.e., 60% of the geographic regions had sample sizes below 100), the data are based on convenience samples of volunteers and likely do not reflect the population under study, and the survey was administered entirely in English. Although age differences in the constructs we examined in the current paper may vary across cultures and geographic regions, it is unclear if these differences can be attributable to statistical power issues, language administration of the survey, or any number of other methodological or conceptual explanations (van Herk et al., 2004; Harzing, 2006; Chopik et al., 2017). A thorough treatment and examination of these issues and cultural differences in perceptions of aging is beyond the scope of the current paper. Nevertheless, we encourage other researchers to examine how variation in aging attitudes might be accounted for by culture-level indicators identified by previous research (e.g., GDP, education, % of older adults, values, and character/personality; McCrae and Terracciano, 2005; Schwartz, 2006; Terracciano and McCrae, 2007; Löckenhoff et al., 2009; Hofstede et al., 2010; Norris and Inglehart, 2011).

Finally, our sample was comprised mostly of young adults on average (*M*_*age*_ = 26.88 years old). Worth noting, even the small percentage of older adults in our sample greatly dwarfed all existing studies on aging perceptions and developmental timing estimates (e.g., there were over 40,000 participants age 50 and over). However, although having such a large sample enabled us to compare people of different ages, it would be ideal to have a more age-balanced, nationally representative sample to provide more precise estimates of all our variables.

## Conclusion

These limitations notwithstanding, the current study advances our knowledge on age differences in age perceptions and developmental timing estimates. The pattern of age differences observed in the current study aligns well with research and theory suggesting that people psychologically distance themselves from older adults by providing younger age perceptions and older developmental transitions.

## Author contributions

WC conducted the analyses, interpreted the data, and drafted the manuscript. RB and DJ interpreted the data and provided critical revisions. DJ constructed the figures. HG provided critical revisions. All authors read and approved the final version of this manuscript.

### Conflict of interest statement

The authors declare that the research was conducted in the absence of any commercial or financial relationships that could be construed as a potential conflict of interest.

## Footnotes

^1^We find the question of how culture might attenuate the effects observed very interesting. Unfortunately, the subsamples of individual countries were too small to make meaningful comparisons. For example, respondents from China (0.3% of the total sample) and South Korea (0.2%) were too small to properly test this question. Aside from the sample size/power issues, the questions were administered entirely in English, and the sample was non-representative of the population from which it was drawn. We expand on the question of testing cultural differences in the section Discussion.

^2^Correlations and descriptive statistics for younger (aged 10–39), middle-aged (aged 40–64), and older adults (aged 65+) are reported in Supplementary Tables 1–3.

^3^There are a few different ways of modeling subjective age in the literature. In the current study, we analyzed the raw ages that participants provided in order to maintain interpretational consistency across models and outcomes. However, previous researchers have also expressed subjective age as a discrepancy score (i.e., chronological age-subjective age; e.g., Weiss and Lang, 2012) and a proportional discrepancy (i.e., chronological age-subjective age/chronological age; e.g., Rubin and Berntsen, 2006). We re-ran the models with these two metrics of subjective age; these results can be found in Supplementary Tables 4, 5 and Supplementary Figures 1, 2. Their interpretation is consistent to what is reported in the main text and in Figure 1. Specifically, as individuals age, they report increasingly younger subjective ages relative to their chronological age. This proportional distortion grew across the lifespan, eventually peaking at just over 20%, which is consistent with previous research (Rubin and Berntsen, 2006).
