# Supplementary material for: Age Differences in Age Perceptions and Developmental Transitions
Source: Front Psychol. 2018 Feb 1;9:67. doi: 10.3389/fpsyg.2018.00067 (PMC5799826; doi:10.3389/fpsyg.2018.00067)
Supplement: Supplementary file 1 [file Table1.DOCX]

| Supplementary Table 1. Correlations and Descriptive Statistics among Primary Study Variables for Young Adults (aged 10-39 years) | | | | | | | | | | |
| --- | --- | --- | --- | --- | --- | --- | --- | --- | --- | --- |
|  | 1 | 2 | 3 | 4 | 5 | 6 | 7 | 8 | 9 | 10 |
| 1.) Gender |  |  |  |  |  |  |  |  |  |  |
| 2.) Age | -.05** |  |  |  |  |  |  |  |  |  |
| 3.) Age Choice | -.01* | .42** |  |  |  |  |  |  |  |  |
| 4.) Subjective Age | -.03** | .53** | .34** |  |  |  |  |  |  |  |
| 5.) Hope to Live | .02** | -.04** | -.02** | -.04* |  |  |  |  |  |  |
| 6.) Perceived Age | -.13** | .67** | .35** | .50** | -.03** |  |  |  |  |  |
| 7.) Childhood-Young Adult Transition | .02** | .08** |  |  |  |  |  |  |  |  |
| 8.) Young Adult-Adult Transition | .02** | .11** |  |  |  |  | .60** |  |  |  |
| 9.) Adult-Middle Age Transition | .08** | .17** |  |  |  |  | .19** | .37** |  |  |
| 10.) Middle Age-Older Adulthood Transition | .11** | .18** |  |  |  |  | .08** | .19** | .63** |  |
| M | -- | 22.30 | 21.87 | 23.13 | 89.50 | 21.56 | 15.62 | 21.87 | 39.41 | 61.74 |
| SD | -- | 5.85 | 6.73 | 8.13 | 16.39 | 5.85 | 3.07 | 4.12 | 7.15 | 9.50 |
| Note. Ns range from 208626 to 418573. All correlations are significant at p < .001. Gender: -1: Male, 1: Female) | | | | | | | |  |  |  |
