# Supplementary material for: Age Differences in Age Perceptions and Developmental Transitions
Source: Front Psychol. 2018 Feb 1;9:67. doi: 10.3389/fpsyg.2018.00067 (PMC5799826; doi:10.3389/fpsyg.2018.00067)
Supplement: Supplementary file 2 [file Table2.DOCX]

| Supplementary Table 2. Correlations and Descriptive Statistics among Primary Study Variables for Middle-aged Adults (aged 40-64 years) | | | | | | | | | | |
| --- | --- | --- | --- | --- | --- | --- | --- | --- | --- | --- |
|  | 1 | 2 | 3 | 4 | 5 | 6 | 7 | 8 | 9 | 10 |
| 1.) Gender |  |  |  |  |  |  |  |  |  |  |
| 2.) Age | .01* |  |  |  |  |  |  |  |  |  |
| 3.) Age Choice | .11** | .38** |  |  |  |  |  |  |  |  |
| 4.) Subjective Age | -.05** | .42** | .30** |  |  |  |  |  |  |  |
| 5.) Hope to Live | .01 | .02* | .02* | -.08** |  |  |  |  |  |  |
| 6.) Perceived Age | -.10** | .73** | .35** | .52** | -.02** |  |  |  |  |  |
| 7.) Childhood-Young Adult Transition | .07** | .05** |  |  |  |  |  |  |  |  |
| 8.) Young Adult-Adult Transition | .07** | .07** |  |  |  |  | .58** |  |  |  |
| 9.) Adult-Middle Age Transition | .12** | .09** |  |  |  |  | .14** | .25** |  |  |
| 10.) Middle Age-Older Adulthood Transition | .18** | .12** |  |  |  |  | .04** | .11** | .53** |  |
| M | -- | 49.49 | 35.94 | 40.22 | 88.98 | 42.40 | 16.35 | 22.85 | 43.69 | 68.03 |
| SD | -- | 6.49 | 10.16 | 8.90 | 11.89 | 8.05 | 3.19 | 4.27 | 6.90 | 8.45 |
| Note. Ns range from 38250 to 76758. All correlations are significant at p < .001. Gender: -1: Male, 1: Female) | | | | | | | |  |  |  |
