# Supplementary material for: Age Differences in Age Perceptions and Developmental Transitions
Source: Front Psychol. 2018 Feb 1;9:67. doi: 10.3389/fpsyg.2018.00067 (PMC5799826; doi:10.3389/fpsyg.2018.00067)
Supplement: Supplementary file 3 [file Table3.DOCX]

| Supplementary Table 3. Correlations and Descriptive Statistics among Primary Study Variables for Older Adults (aged 65+ years) | | | | | | | | | | |
| --- | --- | --- | --- | --- | --- | --- | --- | --- | --- | --- |
|  | 1 | 2 | 3 | 4 | 5 | 6 | 7 | 8 | 9 | 10 |
| 1.) Gender |  |  |  |  |  |  |  |  |  |  |
| 2.) Age | -.05* |  |  |  |  |  |  |  |  |  |
| 3.) Age Choice | .15** | .12** |  |  |  |  |  |  |  |  |
| 4.) Subjective Age | -.13** | .31** | .18** |  |  |  |  |  |  |  |
| 5.) Hope to Live | -.02 | .11** | .05* | -.09** |  |  |  |  |  |  |
| 6.) Perceived Age | -.09** | .48** | .15** | .45** | .01 |  |  |  |  |  |
| 7.) Childhood-Young Adult Transition | .07* | .02 |  |  |  |  |  |  |  |  |
| 8.) Young Adult-Adult Transition | .09** | .02 |  |  |  |  | .60** |  |  |  |
| 9.) Adult-Middle Age Transition | .13** | .06* |  |  |  |  | .18** | .33** |  |  |
| 10.) Middle Age-Older Adulthood Transition | .17** | .03 |  |  |  |  | .10** | .16** | .55** |  |
| M | -- | 69.47 | 47.47 | 53.81 | 90.01 | 60.22 | 16.66 | 23.73 | 46.05 | 70.79 |
| SD | -- | 4.64 | 14.48 | 12.79 | 9.51 | 7.87 | 3.33 | 4.78 | 6.89 | 8.37 |
| Note. Ns range from 2156 to 4413. All correlations are significant at p < .001. Gender: -1: Male, 1: Female) | | | | | | |  |  |  |  |
