# Supplementary material for: Age Differences in Age Perceptions and Developmental Transitions
Source: Front Psychol. 2018 Feb 1;9:67. doi: 10.3389/fpsyg.2018.00067 (PMC5799826; doi:10.3389/fpsyg.2018.00067)
Supplement: Supplementary file 4 [file Table4.DOCX]

| Supplementary Table 4. Regression Results for Subjective Age (Difference Score) | | | | | | | | |  |  |
| --- | --- | --- | --- | --- | --- | --- | --- | --- | --- | --- |
|  | *b* | *SE* | *β* | | *t* | *p* | 95% CI: LB | 95% CI: UB | *Fchange* | *ΔR^2^* |
| Age | .30 | .002 | .43 | | 153.56 | <.001 | .297 | .305 | 23581.67 | .07 |
| Gender | .14 | .02 | .02 | | 8.95 | < .001 | .110 | .172 | 80.12 | .0002 |
| Age^2^ | .01 | < .001 | .29 | | 45.80 | < .001 | .009 | .009 | 2097.18 | .006 |
| Age^3^ | < .001 | < .001 | -.22 | | -36.92 | < .001 | -.0002 | -.0002 | 1362.73 | .004 |
| *F*(4, 249734) = 74867.02, *p* < .001. *R^2^* = .55. | | | | | |  |  |  |  |  |
|  |  |  |  |  | |  |  |  |  |  |
