# Supplementary material for: Age Differences in Age Perceptions and Developmental Transitions
Source: Front Psychol. 2018 Feb 1;9:67. doi: 10.3389/fpsyg.2018.00067 (PMC5799826; doi:10.3389/fpsyg.2018.00067)
Supplement: Supplementary file 5 [file Table5.DOCX]

| Supplementary Table 5. Regression Results for Subjective Age (Proportional Score) | | | | | | | |  |  |
| --- | --- | --- | --- | --- | --- | --- | --- | --- | --- |
|  | *b* | *SE* | *β* | *t* | *p* | 95% CI: LB | 95% CI: UB | *Fchange* | *ΔR^2^* |
| Age | .70 | .002 | .39 | 125.42 | <.001 | .011 | .011 | 15730.97 | .06 |
| Gender | -.14 | .02 | .01 | 6.63 | < .001 | .003 | .006 | 43.93 | .0002 |
| Age^2^ | < .001 | < .001 | -.05 | -6.72 | < .001 | -.0001 | -.00004 | 45.10 | .0002 |
| Age^3^ | < .001 | < .001 | -.05 | -8.09 | < .001 | -.000002 | -.000001 | 65.39 | .0002 |
| *F*(4, 249734) = 74867.02, *p* < .001. *R^2^* = .55. | | | | |  |  |  |  |  |
