# Supplementary figures and images for: Age Differences in Age Perceptions and Developmental Transitions
Source: Front Psychol. 2018 Feb 1;9:67. doi: 10.3389/fpsyg.2018.00067 (PMC5799826; doi:10.3389/fpsyg.2018.00067)

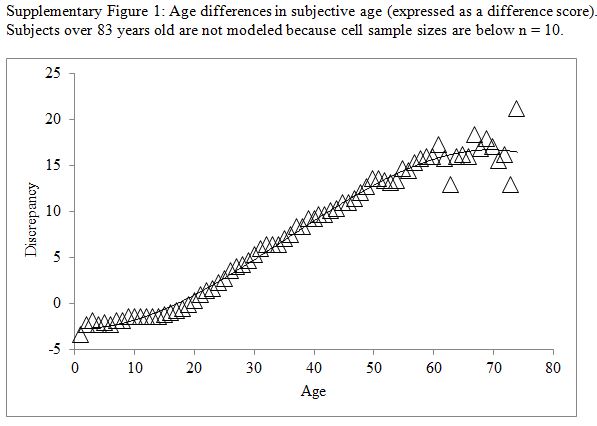

Supplement: Supplementary file 6 [file Image1.JPEG]

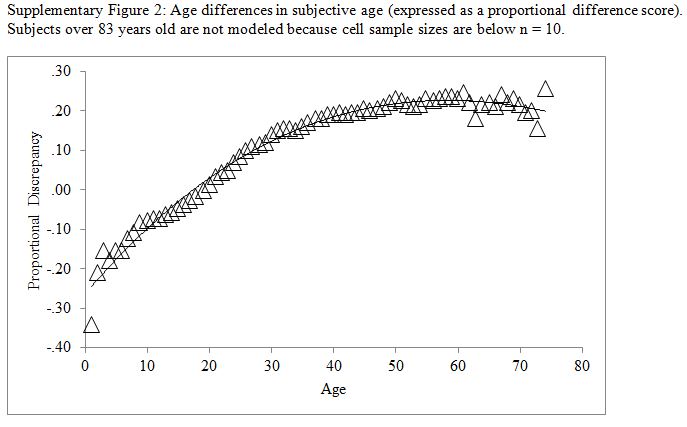

Supplement: Supplementary file 7 [file Image2.JPEG]
